# Investigating Transboundary Spread Patterns and Cluster Characteristics of Lumpy Skin Disease (LSD) Outbreaks in Asia: Levering the Outbreak Data (2019–2023) to Support the LSD Prevention and Control Strategies

**DOI:** 10.1155/tbed/2964021

**Published:** 2025-08-06

**Authors:** Veerasak Punyapornwithaya, Supitchaya Srisawang, Orapun Arjkumpa, Waraphon Phimpraphai, Noppawan Buamithup, Bolortuya Purevsuren, Karma Rinzin, Ronello Abila, Ashish Sutar

**Affiliations:** ^1^Research Center for Veterinary Biosciences and Veterinary Public Health, Faculty of Veterinary Medicine, Chiang Mai University, Chiang Mai 50100, Thailand; ^2^Animal Health Section of the 4th Regional Livestock Office, Department of Livestock Development, Ministry of Agriculture and Cooperatives, Khon Kaen 40260, Thailand; ^3^Department of Veterinary Public Health, Faculty of Veterinary Medicine, Kasetsart University, Bangkok 10900, Thailand; ^4^Bureau of Disease Control and Veterinary Services, Department of Livestock Development, Ministry of Agriculture and Cooperatives, Bangkok 10400, Thailand; ^5^Department of Livestock Development, World Organisation for Animal Health (WOAH) Sub-Regional Representation for South East Asia, Bangkok 10400, Thailand

**Keywords:** Asia, directional trend, disease prevention and control strategies, epidemiology, lumpy skin disease, outbreak, regional, spatiotemporal analysis

## Abstract

Lumpy skin disease (LSD) has rapidly spread across Asia, posing significant threats to livestock industries. This study aims to examine the spatial directional trends and spatiotemporal clusters of LSD outbreaks in South, East, and Southeast Asia from January 2019 to December 2023. Official LSD outbreak data were analyzed using spatial and spatiotemporal models. The standard deviational ellipse (SDE) method was applied to assess directional distribution trends, while space–time permutation (STP) and space–time Poisson models were utilized to identify outbreak clusters. A total of 1385 LSD outbreaks were recorded in the study region during the period. In the Asia region, the directional analysis of all outbreaks revealed trends from the south to the southeast and from the northeast to the southeast, based on the SDE case-weighted and SDE case-unweighted approaches, respectively. Additionally, distinct directional patterns were identified for each subregion. A comparison of the space–time Poisson and STP models showed variations in cluster locations and sizes. The STP model identified primary clusters in India, Bhutan, the Republic of Korea, and Vietnam, whereas the space–time Poisson model highlighted primary clusters in India, Thailand, and Mongolia. Both models detected secondary clusters in various countries including India, Pakistan, Bhutan, China, Mongolia, Nepal, Cambodia, Vietnam, Thailand, Indonesia, Malaysia, Singapore, Sri Lanka, and the Republic of Korea, with the space–time Poisson model identifying larger cluster sizes and the STP model capturing more localized clusters. These findings provide valuable insights into the spatial and temporal dynamics of LSD outbreaks in Asia. The results of this study contribute to a better understanding of LSD epidemiology and offer essential information to support the development of regional and subregional prevention and control strategies.

## 1. Introduction

Lumpy skin disease (LSD), caused by the LSD virus (LSDV), is a significant transboundary disease that primarily affects cattle and buffaloes [1, 2]. Animals infected with LSDV typically exhibit clinical signs such as skin nodules [2, 3]. The morbidity and mortality rates associated with LSD vary depending on several factors, including the health status, age, and breed of the animals. Morbidity generally ranges from 10% to 90%, while mortality typically falls between 1% and 5% [1, 4]. The transmission of LSD is primarily vector-borne, with insect vectors such as flies and ticks playing a crucial role [4, 5]. Typically, LSDV transmission over short distances is driven by insect vectors [6, 7], while long-distance transmission is more commonly associated with the movement of infected animals [8].

LSDV has progressively expanded its geographical range, spreading from Africa to the Middle East and subsequently into Asia [9, 10]. The first reported LSD outbreak in Asia occurred in Bangladesh in 2019, followed by outbreaks in neighboring India. Subsequent outbreaks were reported in China and other Southeast Asian countries, including Vietnam, Laos, Thailand, Malaysia, and Indonesia [11]. These outbreaks have caused significant economic losses, with estimates suggesting that LSD outbreaks in Asia have resulted in losses amounting to approximately 1.45 billion dollars [12]. The severity of the outbreaks has drawn the attention of international organizations concerned with animal health, such as the World Organisation for Animal Health (WOAH) and the Food and Agriculture Organization (FAO). In response, WOAH [13] has initiated cooperative efforts among Southeast Asian nations to develop and implement effective prevention and control strategies at both national and regional levels.

The application of geographical information systems and spatial epidemiological analysis has proven to be a valuable approach in investigating the epidemiology of diseases. Mapping disease outbreaks is particularly beneficial, as it provides a clear illustration of spatial distribution patterns. Beyond the visual insights offered by mapping, identifying the directional distribution trends of outbreak clusters can reveal the spatial pathways of disease spread. This approach, which highlights the mean center, standard deviation, and directional trend, has been effectively demonstrated in numerous studies [14, 15]. Moreover, spatiotemporal analysis helps identify regions with elevated disease incidence, which is crucial for understanding the temporal and spatial dynamics of disease transmission [16]. Spatiotemporal models have been particularly effective in identifying clusters of transboundary diseases, such as foot-and-mouth disease and LSD [17–21].

In Asia, extensive research efforts have been undertaken to investigate the epidemiology of LSD at the national level. Country-specific studies have been conducted in Bangladesh [3, 22–25], India [19, 26–28], Nepal [29], China [30–32], Vietnam [33–35], Myanmar [36], Thailand [37–41], Malaysia [42], and Indonesia [43–45]. However, despite these country-level investigations, studies that examine LSD outbreaks at the continental scale remain limited [46, 47]. Moreover, recent outbreaks reported in Bhutan, Indonesia, the Republic of Korea, and Thailand in 2022 and 2023 [11] have not yet been thoroughly investigated. The epidemiological characteristics of these recent outbreaks may differ from previous findings, making it important to conduct updated studies. Additionally, efforts are currently underway to establish a regional strategy for the prevention and control of LSD [13]. Incorporating recent outbreak data could reveal updated patterns in spatial trends and space–time clusters. Studies that include data up to 2023 would provide valuable information to academics, authorities, and stakeholders, enhancing efforts to prevent and control the disease.

This study aims to examine the spatial directional trends and spatiotemporal clusters of LSD outbreaks across South, East, and Southeast Asia. The findings are expected to offer an updated understanding of the distribution and spatiotemporal clusters of LSD in Asia, providing valuable insights that can help improve regional prevention and control measures for LSD.

## 2. Materials and Methods

### 2.1. Data

Official LSD outbreak data from the World Animal Health Information System (WAHIS) were used and the data management process was reviewed by WOAH SRR SEA. Duplicate records were excluded and outbreak reports with identical geographical coordinates and dates were consolidated into single report, as recommended by Wilhelm and Ward [10]. Data management was performed using functions from the “*tidyverse*” package in R.

The dataset comprised LSD outbreak data from South Asia (Bangladesh, India, Pakistan, Nepal, Bhutan, and Sri Lanka), East Asia (China, Hong Kong, Mongolia, Chinese Taipei, and the Republic of Korea), and Southeast Asia (Myanmar, Vietnam, Laos, Cambodia, Thailand, Malaysia, Singapore, and Indonesia). The final dataset included outbreak location data (latitude and longitude), onset dates (day, month, and year), the number of LSD cases, and the number of susceptible animals, which represented the population at risk.

Cattle density data were sourced from the FAO (https://data.apps.fao.org/catalog//iso/9d1e149b-d63f-4213-978b-317a8eb42d02). This dataset provides gridded estimates of cattle population density in raster format, expressed as the number of cattle per square kilometer at a high spatial resolution.

For spatiotemporal analyses, the data were organized into four scenarios: (1) Scenario OA combined data from South, East, and Southeast Asia; (2) Scenario SA focused on South Asia; (3) Scenario EA examined East Asia; (4) Scenario SEA focused on Southeast Asia. This structure provided both an overall regional perspective and more detailed regional analyses.

### 2.2. Directional Distribution

The directional distribution of LSD outbreaks was analyzed using the standard deviational ellipse (SDE) method. This method calculates the mean center of LSD outbreak locations and measures the standard distance in both the *x*- and *y*-directions from the center. The SDE analysis provides the semimajor and semiminor axes, representing the long and short axes, respectively, along with the rotation angle. The major axis represents the predominant direction of LSD outbreak spread, while its length indicates the extent of spatial distribution. The azimuth reflects the main trend direction, and the ratio of the major to minor axes provides insights into the overall shape of the spatial dispersion [48]. The analysis was performed using the SDE plugin (v3.34.2) in Quantum Geographic Information System (QGIS) version 3.34.11 (www.qgis.org). Both the Yuill and CrimeStat methods were applied using case-weighted and case-unweighted approaches, resulting in four combinations: Yuill case-weighted, Yuill case-unweighted, CrimeStat case-weighted, and CrimeStat case-unweighted, to examine variations in spatial direction and distribution [10].

### 2.3. Space–Time Clustering

Space–time permutation (STP) and space–time Poisson (Poisson) models were employed to identify spatiotemporal clusters of LSD outbreaks. The data included geographical coordinates and the number of cases per location, with the Poisson model also incorporating population-at-risk data. The details and computational formulas for the STP and Poisson models are well-documented in prior studies [41, 48, 49].

In this study, we utilized SaTScan software for spatiotemporal analyses, carefully considering its parameter settings due to their potential impact on the analytical results. While many studies default to standard settings, this approach may not be suitable for all datasets, as it can sometimes produce excessively large clusters that are not informative for specific research purposes [50]. SaTScan has two different approaches in parameter settings, including maximum spatial cluster size (MSCS) and maximum report cluster size (MRCS), which can affect the size of outbreak clusters [51]. Conceptually, the parameter settings of MSCS and MRCS yield different results [41, 51]. The setting of MSCS limits the clusters evaluated by the analysis, whereas the setting of MRCS limits the clusters reported in the output under the advanced output features [50 ]. In this study, we adhered to the guideline against varying the MSCS for spatiotemporal analysis to avoid statistical issues [46]. Hence, this study varied MRCS through advanced parameter settings. It is mentioned that the use of 50% MSCS may yield too large and uninformative clusters for a geographically large area; thus, using 10% MSCS or adding another maximum geographical cluster size (e.g., 200 km) is suggested [50]. In our data, we set the MRCS to 10%, following a previous suggestion [50].

Results from the spatiotemporal analyses were imported into QGIS for map creation. Geographical materials, including vector and raster files (such as the Asia shapefile), were sourced from publicly available websites.

## 3. Results

### 3.1. LSD Outbreak Distribution

From 2019 to 2023, a total of 1385 LSD outbreaks were reported in the study region. In 2019, there were only eight outbreaks, increasing to 68 in 2020. The most significant rise occurred in 2021, with 1108 outbreaks, followed by a notable decline to 61 in 2022. However, an increase was observed in 2023, with 140 outbreaks.

**Figure 1:**
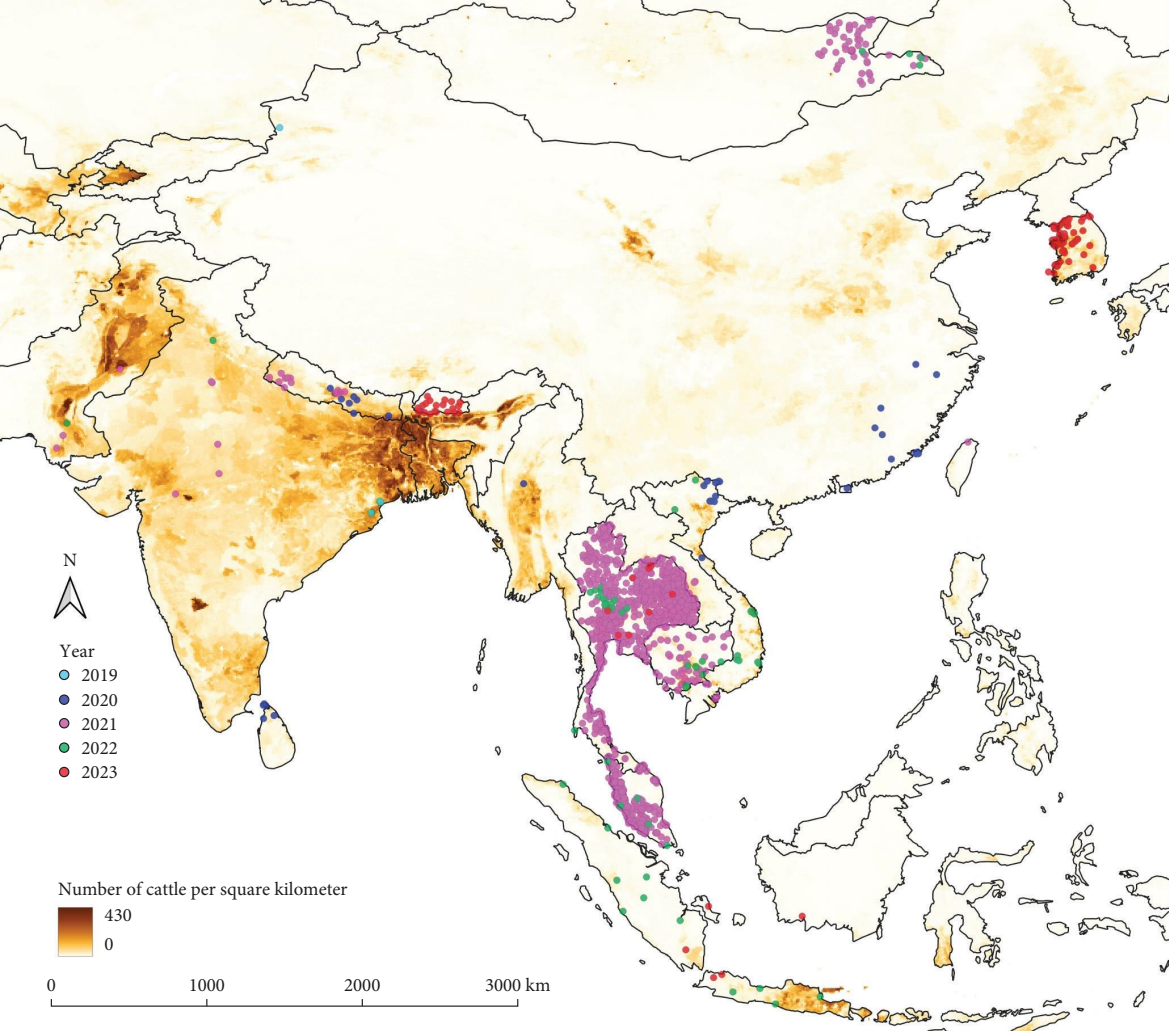
Geographic distribution of lumpy skin disease outbreak locations across South, East, and Southeast Asia from 2019 to 2023, displayed by year. Colored dots represent outbreak locations by year, overlaid on a cattle density map (number of cattle per square kilometer).

Figure 1 illustrates the geographical coordinates of LSD outbreaks by year. Outbreaks in 2019 were primarily concentrated in South Asian countries such as Bangladesh, India, and Nepal, while China reported several outbreaks by 2020. The majority of outbreaks in 2021 occurred in Thailand, whereas outbreaks in 2022 were concentrated in Indonesia. In 2023, most outbreaks were reported in the Republic of Korea and Bhutan.

### 3.2. Directional Distribution Trend Based on SDE

**Figure 2:**
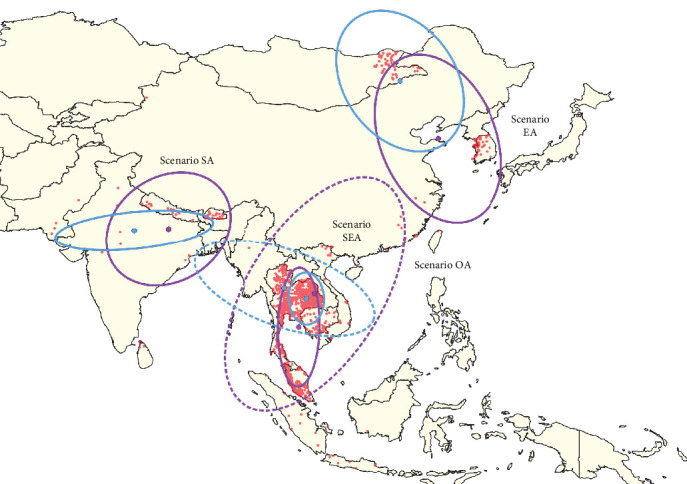
Standard deviational ellipses (SDEs) generated using the Yuill and CrimeStat methods, each applied with case-weighted (blue) and case-unweighted (purple) approaches, illustrating four method-weight combinations for comparison. Yuill and CrimeStat generate the same SDEs; however, the results differ between the case-weighted and case-unweighted approaches. The dashed line represents the SDEs for South, East, and Southeast Asia (Scenario OA), while solid lines represent SDEs for South Asia (Scenario SA), East Asia (Scenario EA), and Southeast Asia (Scenario SEA).

Both the Yuill and CrimeStat methods produced identical SDEs; however, the case-weighted and unweighted approaches yielded different results. In Scenario OA, the case-unweighted approach indicated a clear directional trend from the northeast to the southwest, while the case-weighted analysis revealed a direction from the northwest to the southeast. The SDEs for Scenario SA exhibited a similar directional trend, while Scenarios EA and SEA showed comparable patterns (Figure 2). Notably, the size and shape of the SDEs varied depending on whether a case-weighted or unweighted approach was applied.

### 3.3. Spatiotemporal Clusters

The number of clusters identified by the STP and Poisson models varied across each scenario (Table 1), with notable differences in both the size and time frame of the clusters (Table 2). The STP model consistently identified a greater number of LSD outbreak clusters across all scenarios compared to the Poisson model. However, the Poisson model tended to identify clusters with larger average sizes, particularly in Scenario SEA, where it detected significantly larger clusters. In contrast, the STP model generally produced smaller cluster sizes, with the smallest average cluster size observed in Scenario EA. Overall, the STP model identified more clusters, while the Poisson model focused on fewer but larger clusters.

**Figure 3:**
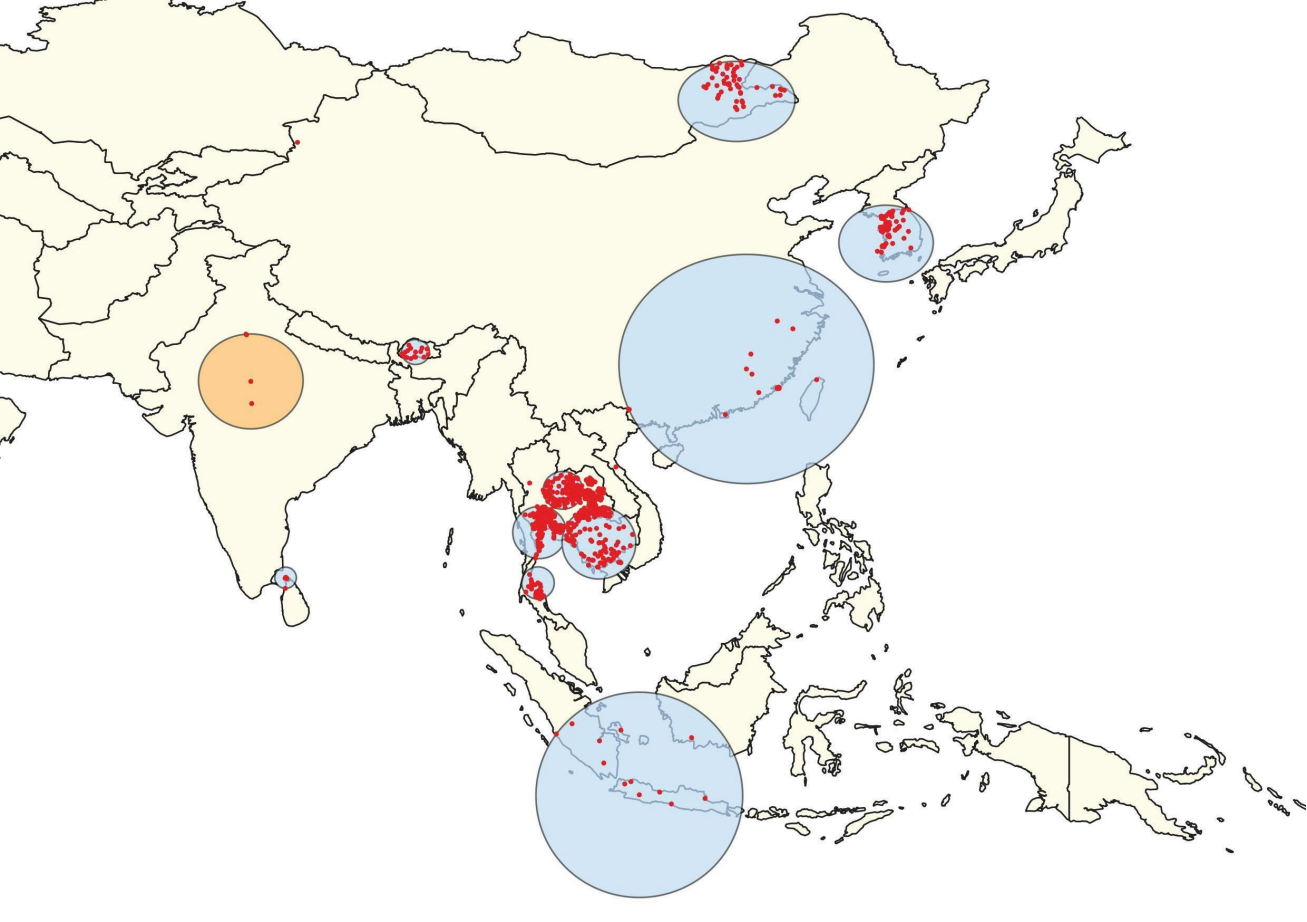
Clusters of lumpy skin disease determined by the space–time permutation model using data from countries in South, East, and Southeast Asia (Scenario OA). The primary cluster is shown in yellow, while secondary clusters are displayed in blue. Red dots represent individual outbreak locations.

**Figure 4:**
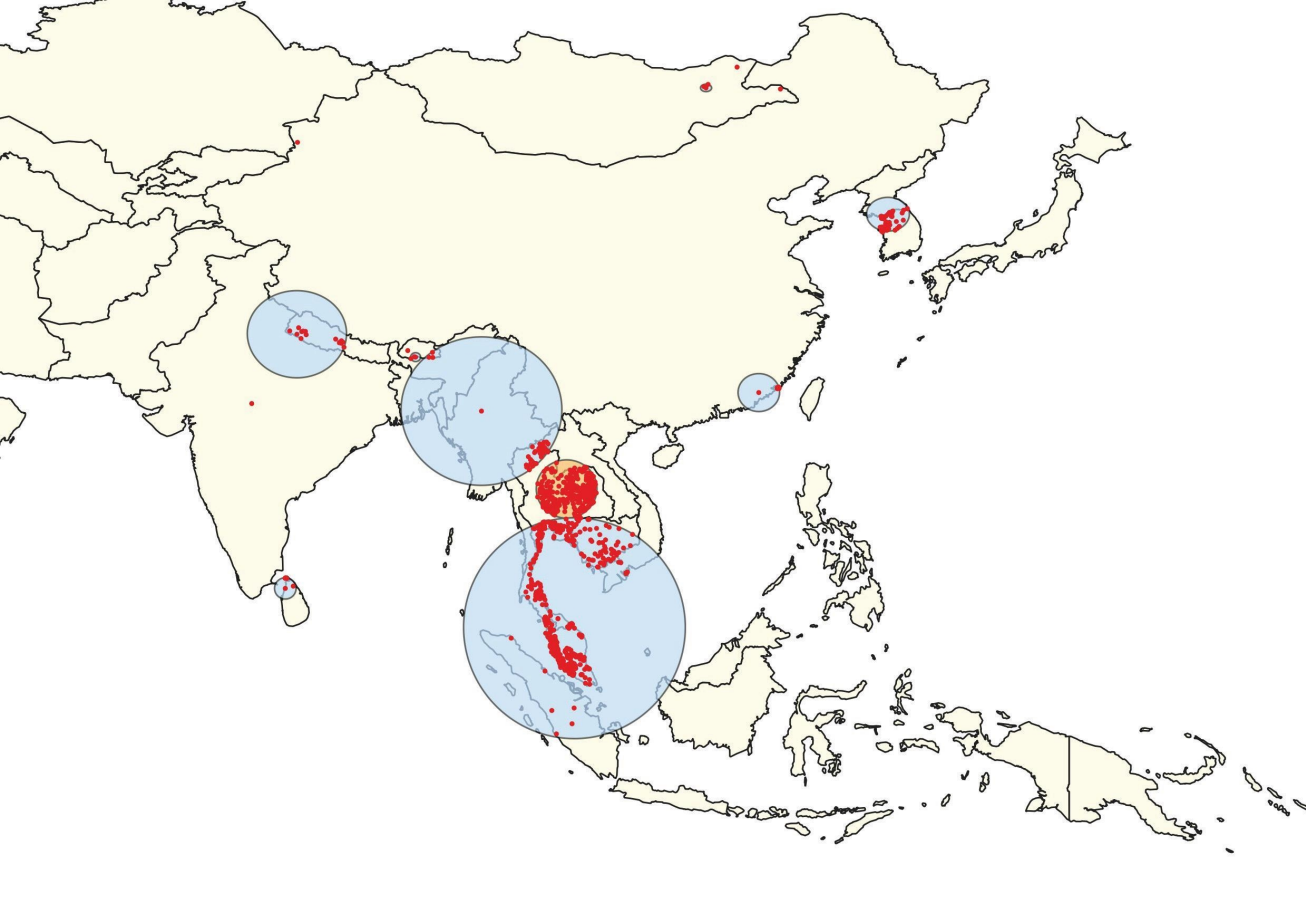
Clusters of lumpy skin disease determined by the space–time Poisson model using data from countries in South, East, and Southeast Asia (Scenario OA). The primary cluster is shown in yellow, while secondary clusters are displayed in blue. Red dots represent individual outbreak locations.

**Figure 5:**
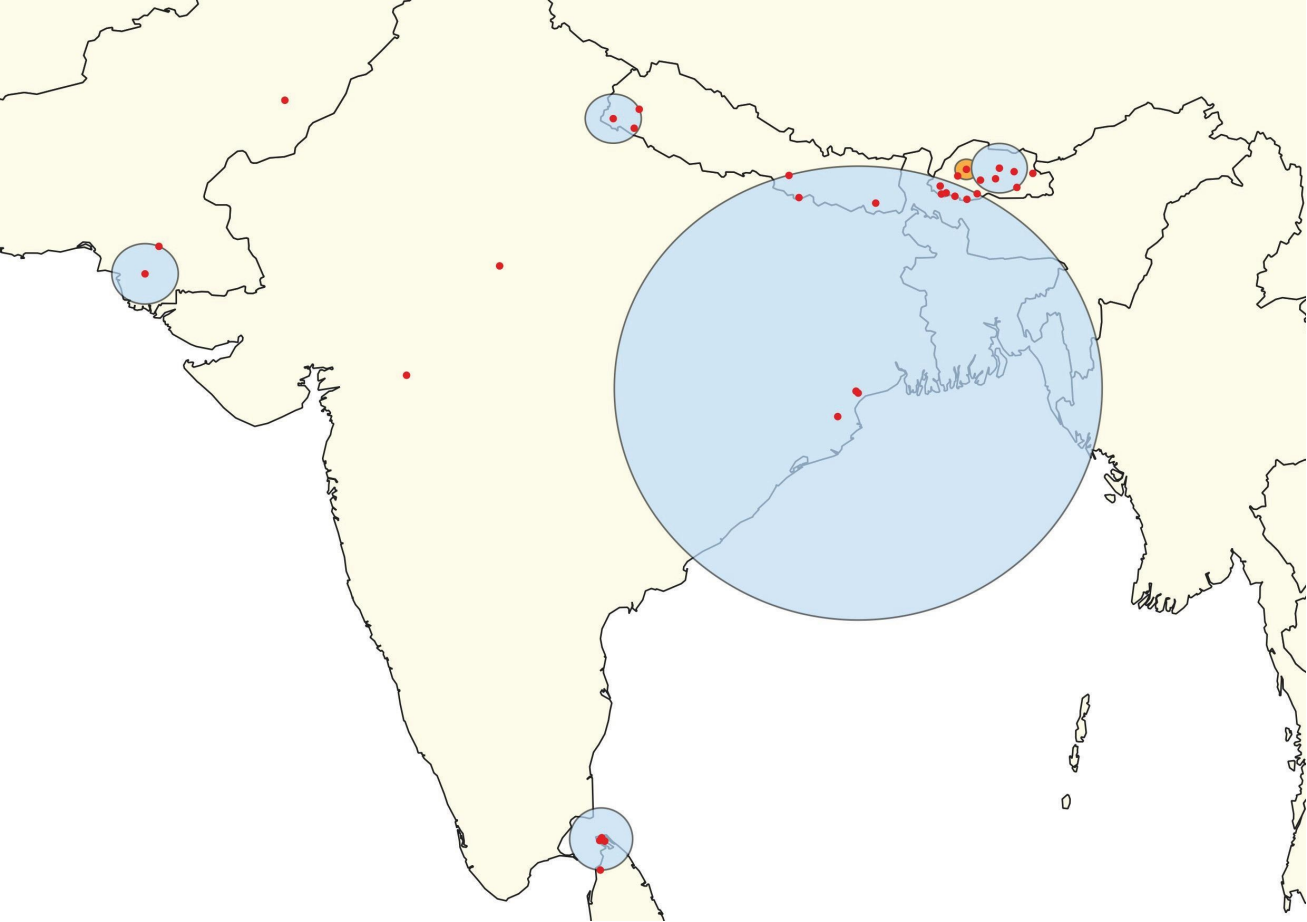
Clusters of lumpy skin disease determined by the space–time permutation model using data from countries in South Asia (Scenario SA). The primary cluster is shown in yellow, while secondary clusters are displayed in blue. Red dots represent individual outbreak locations.

**Figure 6:**
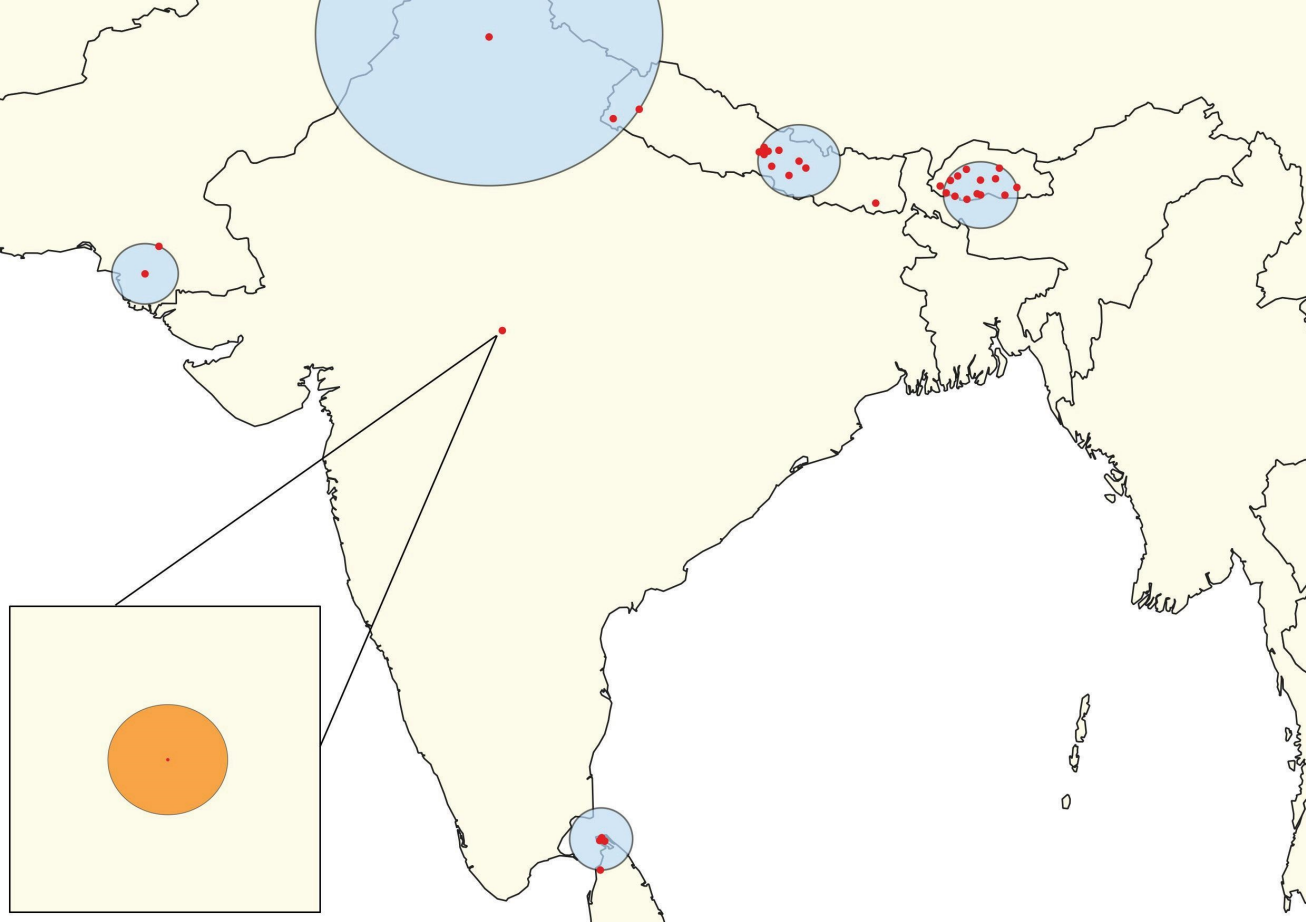
Clusters of lumpy skin disease outbreaks identified using the space–time Poisson model in South Asia (Scenario SA). The primary cluster is highlighted in orange with a zoom-in view displayed in the bottom left corner. Secondary clusters are shown in blue. Red dots represent individual outbreak locations.

**Figure 7:**
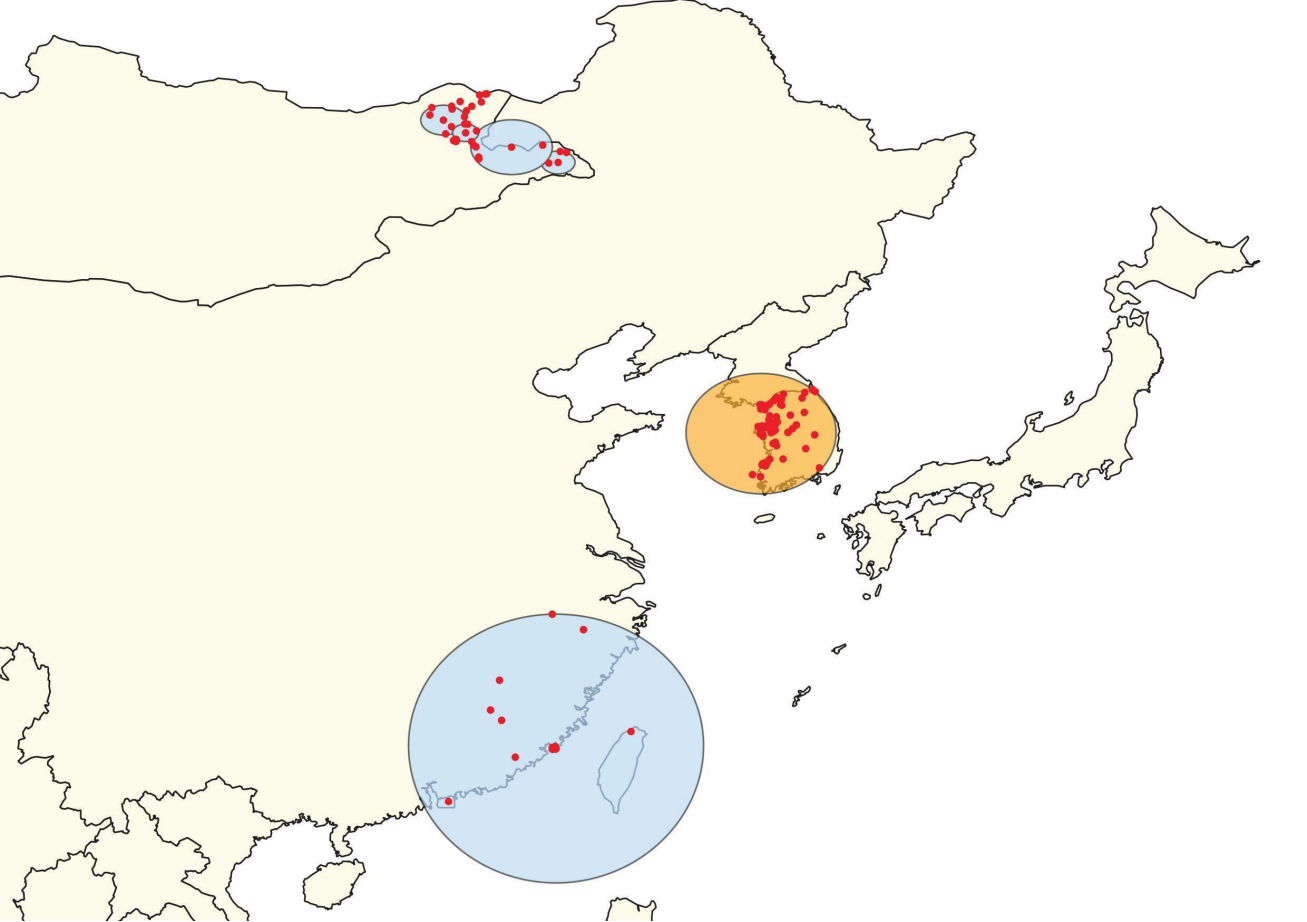
Clusters of lumpy skin disease determined by the space–time permutation model using data from countries in East Asia (Scenario EA). The primary cluster is shown in yellow, while secondary clusters are displayed in blue. Red dots represent individual outbreak locations.

**Figure 8:**
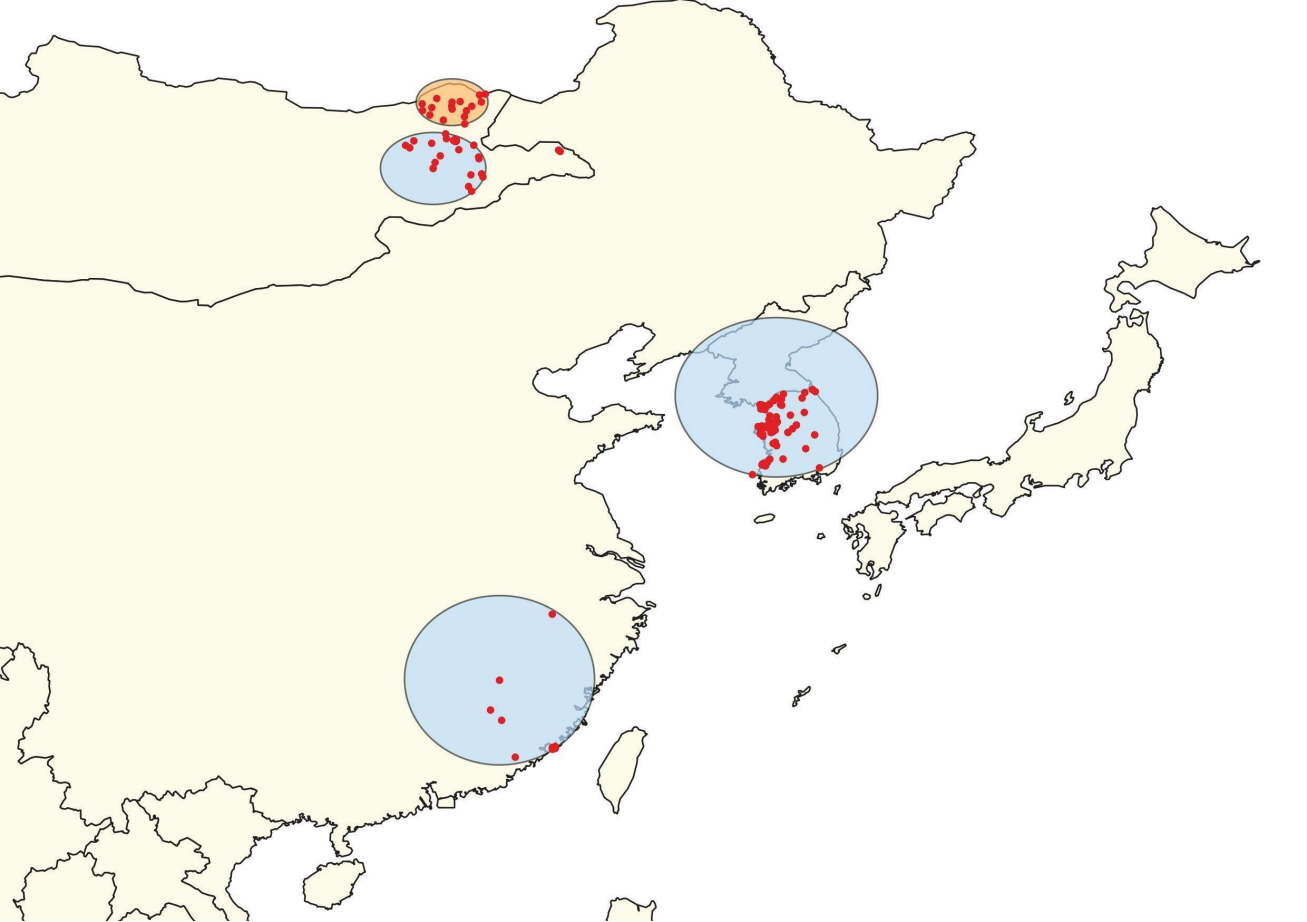
Clusters of lumpy skin disease determined by the space–time Poisson model using data from countries in East Asia (Scenario EA). The primary cluster is shown in yellow, while secondary clusters are displayed in blue. Red dots represent individual outbreak locations.

**Figure 9:**
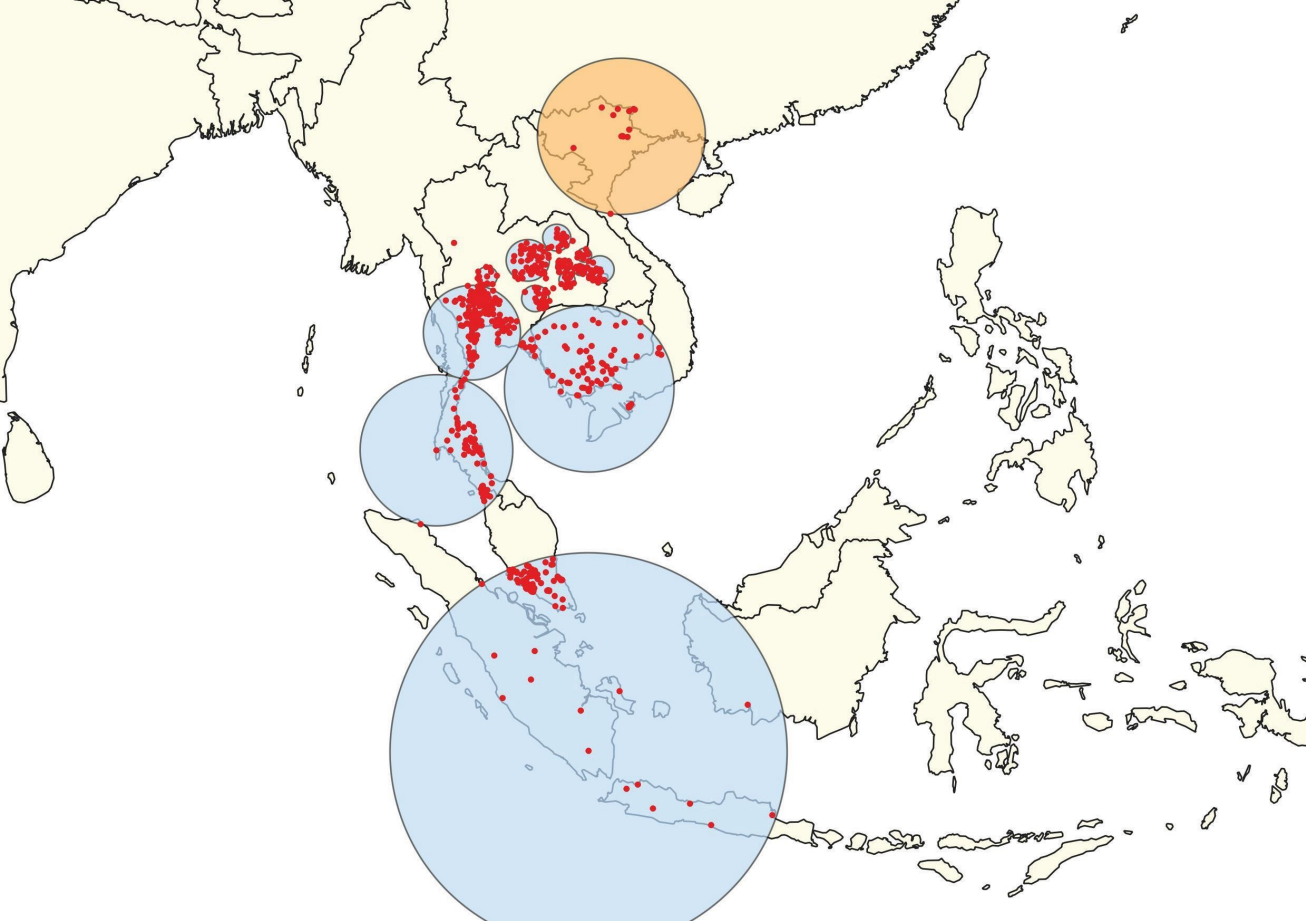
Clusters of lumpy skin disease determined by the space–time permutation model using data from countries in Southeast Asia (Scenario SEA). The primary cluster is shown in yellow, while secondary clusters are displayed in blue. Red dots represent individual outbreak locations.

**Figure 10:**
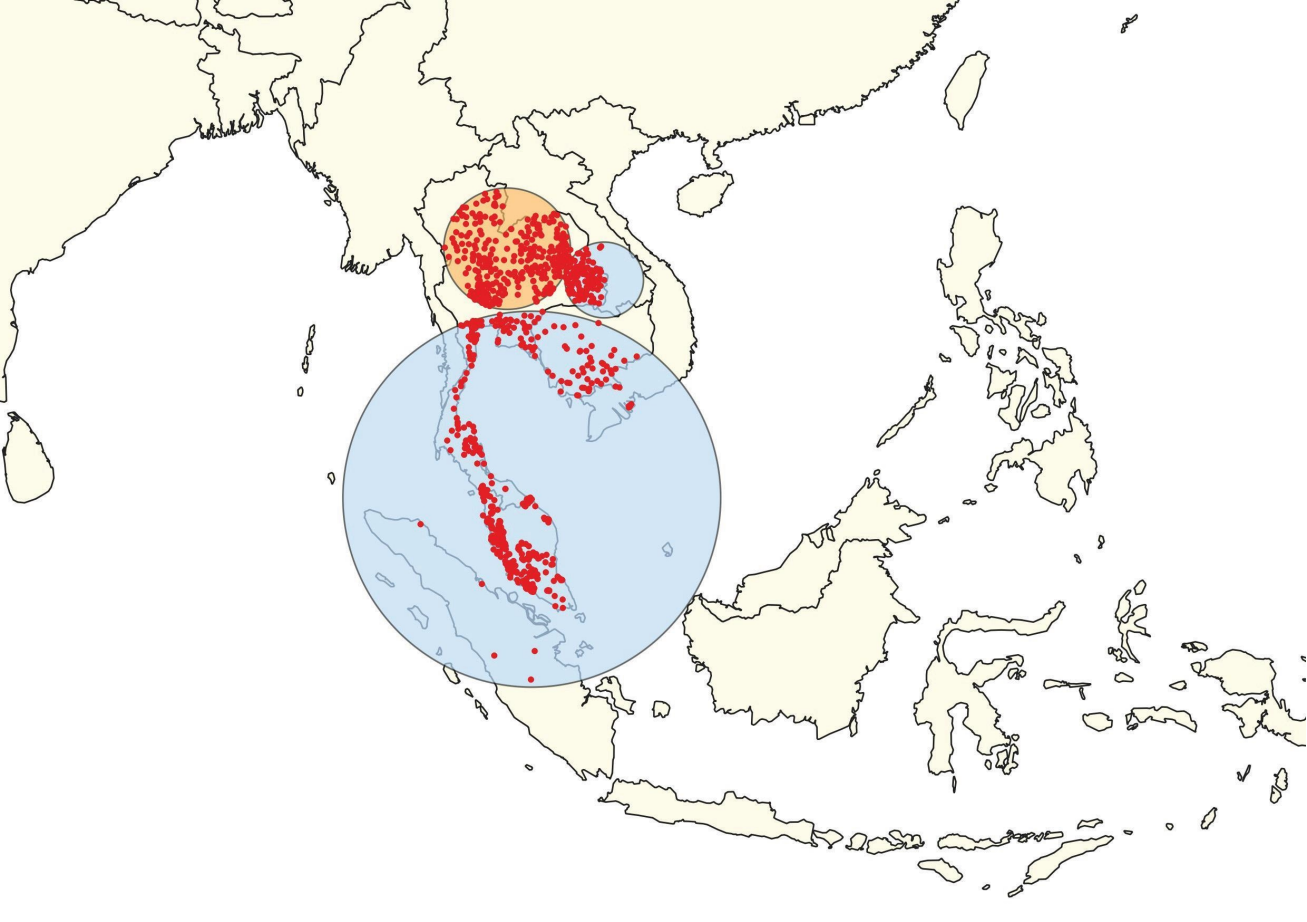
Clusters of lumpy skin disease determined by the space–time Poisson model using data from countries in Southeast Asia (Scenario SEA). The primary cluster is shown in yellow, while secondary clusters are displayed in blue. Red dots represent individual outbreak locations.

In Scenario OA, the STP model identified the primary clusters in India (Figure 3), whereas the Poisson model placed the primary clusters in Thailand (Figure 4). For South Asia (Scenario SA), the STP model identified primary clusters in Bhutan (Figure 5), while the Poisson model found them in India (Figure 6). In East Asia (Scenario EA), the STP model identified the Republic of Korea as the location of the primary cluster (Figure 7), while the Poisson model highlighted Mongolia (Figure 8). In Scenario SEA, the STP model identified Vietnam as the primary cluster location (Figure 9), while the Poisson model indicated Thailand (Figure 10).

The secondary clusters identified by both models spanned a wide geographic area, covering multiple countries across different scenarios (Figures 3–10). The full list of secondary clusters for each scenario is provided in Table 3. For comparison, Table 3 also outlines the countries that formed part of the primary clusters for each method and model. Additionally, the details of the secondary clusters for each model are provided in Tables S1–S8.

## 4. Discussion

LSD outbreaks have significantly impacted multiple countries across Asia, causing substantial economic losses in the region. A comprehensive understanding of the epidemiology of LSD is essential for developing effective prevention and control strategies at both national and regional levels. This study analyzed LSD outbreaks at both the regional and subregional levels by investigating the directional trends and spatiotemporal patterns of the disease based on official outbreak reports.

In Scenario OA, the SDEs revealed a directional trend extending from central Myanmar toward the eastern and northeastern regions of Thailand when employing the case-weighted approach. In contrast, the SDEs indicated a different trajectory originating from eastern China, passing through Vietnam and into Thailand, oriented from the northeast to the southeast. Despite the differences in directionality, both SDEs suggest that the distribution of LSD outbreaks is predominantly directed toward Thailand, although from varied origins. This finding is particularly relevant, as Thailand reported the highest number of LSD cases during the study period, which strongly influenced the results, especially under the case-weighted approach. Similarly, in Scenarios EA and SEA, the SDEs displayed comparable directional trends, although the size and location of the ellipses varied, reflecting the impact of the different methodologies applied. As discussed earlier, these findings provide valuable insights from multiple perspectives, allowing for interpretations that account for both the inclusion and exclusion of case numbers. Notably, the results from Scenario SEA are consistent with the findings of the previous study [10], as both studies identified the SDEs within Thailand. Furthermore, phylogenetic analyses from several studies have demonstrated that LSDV strains from China, Vietnam, and Thailand are genetically similar [39, 40], suggesting a possible role of animal movement along livestock trade pathways [12], which correspond to the directional trends identified in this study, in facilitating the transboundary spread of LSDV. Following the outbreaks in Thailand, subsequent LSD outbreaks were reported in Indonesia, which is located further south in Southeast Asia. Laboratory findings have also shown that LSDV strains isolated in Indonesia are genetically similar to those found in Vietnam and Thailand [44]. Given the alignment of directional trends with the dissemination of genetically similar LSDV strains, it is essential to strengthen disease control measures at national borders to mitigate transboundary transmission [12, 13].

Previous studies on LSD outbreaks in Asia have typically utilized default or modified parameter settings. Notably, when using default settings in various spatiotemporal models, LSD outbreak clusters tend to cover relatively large areas [52]. This approach can obscure smaller and more localized outbreaks that warrant closer examination. Additionally, prior research has indicated that large clusters are often less meaningful for specific research objectives, recommending that parameter settings be adjusted to match the specific goals of the analysis [50]. In past studies on LSD outbreaks in Asia, the maximum spatial window was set to 1000 km, with the maximum temporal window covering 50% of the study period [17]. In contrast, a study focusing on Southeast Asia has set the MSCS to 20% of the population at risk, with a maximum temporal cluster size of 50% [10]. The clusters identified in this study varied in size, time frame, and location compared to those reported in earlier studies by Li et al. [17] and Wilhelm and Ward [10]. However, some clusters were situated in the same countries as those identified in previous research. Notably, the primary clusters identified by the STP and Poisson models in this study had radii of less than 500 km, which is smaller than those reported in previous studies, where eight out of nine spatiotemporal clusters had radii greater than 500 km [17]. Technically, this finding illustrates the influence of parameter settings, emphasizing the importance of clearly defining objectives that specify relevant parameters. Additionally, given that there is no one-size-fits-all setting, if the aim is to explore results based on different parameter configurations, the author of SaTScan recommends varying the MRCS instead of the MSCS [51].

The identification of primary and secondary clusters across multiple countries highlights important aspects of LSD epidemiology in the region. Primary clusters were largely identified in India and Thailand, underscoring their significant role in the spread of LSD during the study period. Secondary clusters were commonly found in countries like Bhutan, China, Indonesia, the Republic of Korea, Myanmar, Vietnam, Bangladesh, India, and Sri Lanka, indicating ongoing disease transmission and localized outbreaks. Clusters confined to individual countries suggest that local factors, such as in-country animal movement and the presence of insect vectors, may play a significant role in transmission [7, 43, 53]. On the other hand, clusters spanning multiple countries, particularly in Southeast Asia, emphasize the need for cross-border coordination due to shared transmission risks, often driven by animal movement across borders [12]. The presence of outbreak clusters both within single countries and across multiple nations emphasizes the importance of comprehensive prevention and control strategies. Efforts should focus on both national-level interventions and regional cooperation. For the regional aspect, controlling animal movement at national borders is critical for preventing cross-border transmission and limiting the further spread of LSD across the region.

Several limitations need to be considered when interpreting the results of this study. One important issue is the potential for reporting bias, which has been previously mentioned in the study using WOAH outbreak data [10]. This bias likely arises from variations in how frequently different countries report outbreaks, as well as differences in reporting systems and policies. Some countries may report outbreaks more consistently, while others may report less frequently or less comprehensively, which could influence the spatial and temporal distribution patterns observed. Another limitation relates to the parameter settings used in spatial and spatiotemporal analyses. These settings can significantly influence the results, and they should be carefully adjusted based on the specific objectives of each investigation.

Future research should follow up on these findings by incorporating epidemiological modeling that accounts for environmental factors such as climate, land use, and vector populations. Such models would provide a more comprehensive understanding of the factors driving the spread of LSD and help inform more effective prevention and control strategies.

In terms of implications, this study identifies the direction of LSD transmission and the outbreak clusters at a regional level, reinforcing the importance of addressing LSD as a regional concern, as emphasized by both WOAH [13] and FAO [12]. Understanding the spatial dynamics of LSD outbreaks can enhance surveillance efforts and inform targeted interventions, ensuring efficient resource allocation to high-risk areas. Furthermore, the findings provide valuable insights to support the development of an ASEAN LSD prevention and control strategy, coordinated by WOAH [13], which is anticipated to be endorsed in 2024.

## 5. Conclusion

This study offers valuable insights into the characteristics of LSD outbreaks across Asia, focusing on directional distribution trends and spatiotemporal clusters identified from regional outbreak data. The examination of these trends and clusters at both overall and subregional levels provides a comprehensive understanding of the spatial and temporal distribution of LSD outbreaks. Furthermore, the findings emphasize the importance of prioritizing national-level cooperations, particularly in regulating animal movement at national borders. Coordinated efforts are essential to prevent cross-border transmission and control the spread of LSD throughout the region. Ultimately, the insights from this study have the potential to support the development of effective regional and subregional prevention and control strategies.

## Figures and Tables

**Table 1:** Number of lumpy skin disease outbreak clusters identified using space–time permutation (STP) and space–time Poisson models for South, East, and Southeast Asia (Scenario OA); South Asia (Scenario SA); East Asia (Scenario EA); Southeast Asia (Scenario SEA).

Model	Scenario	Number of clusters^a^	Average cluster size (km)
SPT	OA	23	182.76
	SA	10	102.86
	EA	14	80.24
	SEA	20	138.26

Poisson	OA	15	180.44
	SA	8	103.41
	EA	6	162.16
	SEA	3	454.53

^a^The number includes both one primary cluster and the secondary clusters.

**Table 2:** Primary lumpy skin disease outbreak clusters identified using the space–time permutation (STP) and space–time Poisson (Poisson) models for South, East, and Southeast Asia (Scenario OA); South Asia (Scenario SA); East Asia (Scenario EA); Southeast Asia (Scenario SEA).

Model	Scenario	Coordinates/radius (km)	Time frame	Number of cases	Expected cases	Relative risk^a^	LLR^b^
STP	OA	(24.971000 N, 77.363000 E)/409.58	2021/8/1 to 2021/8/31	29,640	1875.50	—	54,853.23
	EA	(36.673316 N, 126.418322 E)/259.90	2023/10/1 to 2023/11/30	313	30.39	—	460.24
	SA	(27.541500 N, 89.799100 E)/30.06	2023/4/1 to 2023/4/30	2783	181.70	—	5046.35
	SEA	(21.640200 N, 106.218200 E)/371.78	2020/10/1 to 2020/12/31	12,597	376.04	—	32,192.34

Poisson	OA	(16.613333 N, 101.919722 E)/251.45	2021/4/1 to 2022/3/31	200,141	29,765.14	11.98	255,502.84
	EA	(49.550006 N, 114.404806 E)/100.63	2021/8/1 to 2021/9/30	1264	280.89	6.76	1104.49
	SA	(23.250700 N, 77.434900 E)/<1	2021/8/1 to 2021/8/31	28,650	2790.05	21.91	49,441.01
	SEA	(16.825650 N, 101.320853 E)/288.73	2021/4/1 to 2022/3/31	193,160	33,149.07	11.30	227,611.04

^a^Relative risk from space–time permutation model.

^b^Log likelihood ratio with *p* < 0.001.

**Table 3:** Countries located within primary and secondary clusters identified using the space–time permutation (STP) and space–time Poisson (Poisson) models under each scenario, including South, East, and Southeast Asia (Scenario OA); South Asia (Scenario SA); East Asia (Scenario EA); Southeast Asia (Scenario SEA).

Model	Scenario	Country located within primary cluster	Country located within secondary cluster
STP	OA	India	Bhutan, Cambodia, China, Chinese Taipei, Hong Kong, India, Indonesia, Republic of Korea, Mongolia, Sri Lanka, Thailand, Vietnam
	SA	Bhutan	Bhutan, India, Nepal, Pakistan, Sri Lanka
	EA	The Republic of Korea	China, Chinese Taipei, Hong Kong, Republic of Korea, Mongolia
	SEA	Vietnam	Cambodia, Indonesia, Malaysia, Singapore, Thailand, Vietnam

Poisson	OA	Thailand	Bhutan, Cambodia, China, India, Indonesia, Republic of Korea, Malaysia, Mongolia, Myanmar, Nepal, Singapore, Sri Lanka, Thailand, Vietnam
	SA	India	Bhutan, India, Nepal, Pakistan, Sri Lanka
	EA	Mongolia	China, Republic of Korea, Mongolia
	SEA	Thailand	Cambodia, Indonesia, Laos, Malaysia, Singapore, Thailand, Vietnam

## Data Availability

The data used in this study are available from the World Animal Health Information System (WAHIS) at https://wahis.woah.org.

## Supporting Information

Supporting Information Table S1: Clusters determined by the space–time permutation model for South, East, and Southeast Asia data. Table S2: Clusters determined by the space–time Poisson model for South, East, and Southeast Asia data. Table S3: Clusters determined by the space–time permutation model for South Asia data. Table S4: Clusters determined by the space–time Poisson model for South Asia data. Table S5: Clusters determined by the space–time permutation model for East Asia data. Table S6: Clusters determined by the space–time Poisson model for East Asia data. Table S7: Clusters determined by the space–time permutation model for Southeast Asia data. Table S8:Clusters determined by the space–time Poisson model for Southeast Asia data.
