# Supplementary material for: Investigating Transboundary Spread Patterns and Cluster Characteristics of Lumpy Skin Disease (LSD) Outbreaks in Asia: Levering the Outbreak Data (2019–2023) to Support the LSD Prevention and Control Strategies
Source: Transbound Emerg Dis. 2025 Aug 6;2025:2964021. doi: 10.1155/tbed/2964021 (PMC12349989; doi:10.1155/tbed/2964021)
Supplement: Supporting Information — Table S1: Clusters determined by the space–time permutation model for South, East, and Southeast Asia data. Table S2: Clusters determined by the space–time Poisson model for South, East, and Southeast Asia data. Table S3: Clusters determined by the space–time permutation model for South Asia data. Table S4: Clusters determined by the space–time Poisson model for South Asia data. Table S5: Clusters determined by the space–time permutation model for East Asia data. Table S6: Clusters determined by the space–time Poisson model for East Asia data. Table S7: Clusters determined by the space–time permutation model for Southeast Asia data. Table S8:Clusters determined by the space–time Poisson model for Southeast Asia data. [file 2964021.f1.pdf]

Supplementary Table 1. Clusters determined by space-time permutation model for South, East and South East Asia data

Cluster	Coordinates / radius (km)	Time frame	Number of cases	Expected cases	LLR*	P-value
1	(18.323200 N, 105.753400 E) / < 1 km	2020/12/1 to 2020/12/31	11521	270.50	32102.27	< 0.001
2	(16.166710 N, 103.612595 E) / 44.96 km	2021/4/1 to 2021/4/30	32730	8420.06	20750.02	< 0.001
3	(15.491109 N, 103.887625 E) / 36.88 km	2021/3/1 to 2021/3/31	8562	343.94	19374.68	< 0.001
4	(15.129047 N, 103.299063 E) / < 1 km	2021/7/1 to 2021/7/31	6295	197.01	15747.51	< 0.001
5	(27.254300 N, 90.173500 E) / 107.43 km	2023/3/1 to 2023/3/31	4733	137.81	12164.31	< 0.001
6	(16.454660 N, 104.587736 E) / 29.79 km	2021/5/1 to 2021/5/31	47329	22427.60	11119.51	< 0.001
7	(7.148788 S, 107.573070 E) / 886.18 km	2022/12/1 to 2022/12/31	2712	18.35	10861.70	< 0.001
8	(17.074690 N, 99.045670 E) / < 1 km	2021/11/1 to 2021/11/30	2517	14.55	10475.10	< 0.001
9	(16.510610 N, 101.626988 E) / 163.37 km	2021/5/1 to 2021/5/31	45410	22655.99	9382.21	< 0.001
10	(14.943823 N, 103.060775 E) / < 1 km	2021/4/1 to 2021/4/30	9536	1670.35	8810.05	< 0.001
11	(15.925540 N, 104.447450 E) / 27.42 km	2021/4/1 to 2021/4/30	9915	2156.78	7428.38	< 0.001
12	(13.231775 N, 99.813307 E) / 225.19 km	2021/6/1 to 2021/6/30	10682	2930.65	6125.97	< 0.001
13	(15.556740 N, 102.985880 E) / < 1 km	2021/6/1 to 2021/6/30	4642	538.56	5912.63	< 0.001
14	(12.418000 N, 104.435160 E) / 311.20 km	2021/5/1 to 2021/5/31	36084	19928.59	5547.87	< 0.001
15	(46.725961 N, 115.136656 E) / 345.76 km	2021/9/1 to 2021/9/30	1418	13.91	5155.06	< 0.001

16	(17.159282 N, 104.132534 E) / < 1 km	2021/6/1 to 2021/6/30	3525	408.97	4486.72	< 0.001
17	(9.313889 N, 99.690278 E) / 138.69 km	2021/10/1 to 2021/10/31	871	4.26	3768.83	< 0.001
18	(17.190793 N, 103.751251 E) / 34.48 km	2021/5/1 to 2021/5/31	15828	7507.93	3556.82	< 0.001
19	(35.682438 N, 126.760238 E) / 331.18 km	2023/10/1 to 2023/10/31	215	0.13	1371.01	< 0.001
20	(25.936111 N, 115.901111 E) / 989.43 km	2020/10/1 to 2020/10/31	202	0.31	1106.96	< 0.001
21	(9.715170 N, 80.066850 E) / 91.93 km	2020/9/1 to 2020/9/30	81	0.038	540.65	< 0.001
22	(43.544250 N, 80.993610 E) / < 1 km	2019/8/1 to 2019/8/31	65	0.019	463.71	< 0.001

Supplementary Table 2. Clusters determined by space-time Poisson model for South, East and South East Asia data

Cluster	Coordinates / radius (km)	Time frame	Number of cases	Expected cases	Relative risk	LLR*	P-value
1	(23.250700 N, 77.434900 E) / < 1 km	2021/8/1 to 2021/8/31	28650	3833.05	7.95	33570.68	< 0.001
2	(5.796809 N, 102.528977 E) / 953.19 km	2021/5/1 to 2021/10/31	29721	9008.55	3.48	15298.16	< 0.001
3	(27.365200 N, 89.567900 E) / < 1 km	2023/4/1 to 2023/4/30	1982	564.69	3.52	1073.67	< 0.001
4	(22.663549 N, 95.299698 E) / 640.76 km	2021/4/1 to 2021/8/31	1805	536.66	3.37	922.97	< 0.001
5	(28.636500 N, 80.947900 E) / 376.19 km	2020/7/1 to 2021/7/31	2431	979.47	2.49	760.88	< 0.001
6	(47.688111 N, 118.546250 E) / < 1 km	2021/9/1 to 2021/9/30	355	122.72	2.89	144.87	< 0.001
7	(49.390500 N, 115.175800 E) / < 1 km	2021/8/1 to 2021/8/31	301	103.12	2.92	124.61	< 0.001
8	(26.859100 N, 90.178100 E) / 38.43 km	2023/2/1 to 2023/2/28	2062	1562.61	1.32	72.74	< 0.001
9	(24.104600 N, 116.863300 E) / 163.70 km	2020/6/1 to 2020/7/31	100	22.36	4.47	72.17	< 0.001
10	(36.571380 N, 126.491755 E) / 12.68 km	2023/10/1 to 2023/10/31	51	7.11	7.18	56.60	< 0.001
11	(43.544250 N, 80.993610 E) / < 1 km	2019/8/1 to 2019/8/31	65	14	4.64	48.79	< 0.001
12	(47.777278 N, 112.761528 E) / 32.34 km	2021/9/1 to 2021/9/30	76	22.86	3.32	38.15	< 0.001
13	(37.959702 N, 126.908441 E) / 145.96 km	2023/11/1 to 2023/11/30	51	19.26	2.65	17.91	< 0.001
14	(8.888179 N, 80.046110 E) / 91.93 km	2020/9/1 to 2020/9/30	63	29.50	2.14	14.30	< 0.001

Supplementary Table 3. Clusters determined by space-time permutation model for South Asia data

Cluster	Coordinates / radius (km)	Time frame	Number of cases	Expected cases	LLR*	P-value
1	(28.892220 N, 80.389790 E) / 72.68 km	2021/7/1 to 2021/7/31	1696	44.88	4530.06	< 0.001
2	(21.585458 N, 86.916670 E) / 671.36 km	2023/1/1 to 2023/1/31	3112	366.38	3971.38	< 0.001
3	(27.573200 N, 90.677800 E) / 73.22 km	2023/3/1 to 2023/3/31	2328	249.17	3157.20	< 0.001
4	(22.060500 N, 74.882900 E) / < 1 km	2021/9/1 to 2021/9/30	748	8.90	2579.48	< 0.001
5	(27.434900 N, 91.569500 E) / < 1 km	2023/5/1 to 2023/5/31	631	6.15	2300.53	< 0.001
6	(9.715170 N, 80.066850 E) / 91.93 km	2020/9/1 to 2020/9/30	81	0.29	376.92	< 0.001
7	(24.759830 N, 67.915350 E) / 89.34 km	2021/12/1 to 2021/12/31	55	0.047	333.99	< 0.001
8	(24.971000 N, 77.363000 E) / < 1 km	2021/8/1 to 2021/8/31	966	443.55	231.56	< 0.001
9	(29.381110 N, 71.640230 E) / < 1 km	2021/10/1 to 2021/10/31	5	0.00039	42.34	< 0.001

Supplementary Table 4. Clusters determined by space-time Poisson models for South Asia data

Cluster	Coordinates / radius (km)	Time frame	Number of cases	Expected cases	Relative risk	LLR*	P-value
1	(26.859100 N, 90.178100 E) / 98.30 km	2023/1/1 to 2023/4/30	12135	3741.41	3.94	6670.53	< 0.001
2	(31.066530 N, 77.081050 E) / 440.04 km	2021/7/1 to 2021/7/31	1690	116.53	14.96	2970.55	< 0.001
3	(27.759591 N, 85.339865 E) / 107.68 km	2020/7/1 to 2021/9/30	896	240.92	3.77	526.01	< 0.001
4	(27.098000 N, 89.101100 E) / < 1 km	2023/3/1 to 2023/3/31	851	299.92	2.87	339.39	< 0.001
5	(9.715170 N, 80.066850 E) / 91.93 km	2020/9/1 to 2020/9/30	81	6.91	11.75	125.38	< 0.001
6	(26.642475 N, 87.383367 E) / < 1 km	2020/6/1 to 2020/6/30	500	310.85	1.61	48.84	< 0.001
7	(24.759830 N, 67.915350 E) / 89.34 km	2021/12/1 to 2021/12/31	55	12.98	4.24	37.42	< 0.001

Supplementary Table 5. Clusters determined by space-time permutation model for East Asia data

Cluster	Coordinates / radius (km)	Time frame	Number of cases	Expected cases	LLR*	P-value
1	(24.441660 N, 118.447590 E) / 580.05 km	2020/6/1 to 2021/4/30	229	16.27	400.10	< 0.001
2	(43.544250 N, 80.993610 E) / < 1 km	2019/8/1 to 2019/8/31	65	1.31	190.70	< 0.001
3	(49.390500 N, 115.175800 E) / < 1 km	2021/8/1 to 2021/8/31	301	90	159.66	< 0.001
4	(48.102756 N, 114.568522 E) / 9.19 km	2021/10/1 to 2021/11/30	98	7.96	157.21	< 0.001
5	(49.866389 N, 115.699444 E) / 4.40 km	2021/8/1 to 2021/8/31	240	71.76	126.08	< 0.001
6	(47.209248 N, 118.529452 E) / 49.46 km	2021/10/1 to 2022/8/31	58	4.23	98.56	< 0.001
7	(48.852656 N, 114.068502 E) / 64.45 km	2021/8/1 to 2021/8/31	246	90	95.30	< 0.001
8	(47.803000 N, 116.717000 E) / 118.35 km	2022/6/1 to 2022/7/31	35	1.22	83.98	< 0.001
9	(49.829278 N, 115.474847 E) / < 1 km	2021/9/1 to 2021/9/30	292	128.43	80.67	< 0.001
10	(48.360028 N, 114.931028 E) / 37.56 km	2021/9/1 to 2021/9/30	221	97.20	60.20	< 0.001
11	(49.558417 N, 115.544944 E) / < 1 km	2021/8/1 to 2021/8/31	95	28.41	48.79	< 0.001
12	(49.574472 N, 114.719639 E) / < 1 km	2021/9/1 to 2021/9/30	86	37.83	22.82	< 0.001
13	(49.217427 N, 114.964544 E) / < 1 km	2021/9/1 to 2021/9/30	72	31.67	19.06	< 0.001

Supplementary Table 6. Clusters determined by space-time Poisson model for East Asia data

Cluster	Coordinates / radius (km)	Time frame	Number of cases	Expected cases	Relative risk	LLR*	P-value
1	(47.636288 N, 118.610740 E) / 7.51 km	2021/9/1 to 2021/9/30	355	22.29	17.78	667.90	< 0.001
2	(38.085883 N, 127.015455 E) / 344.06 km	2023/10/1 to 2023/11/30	312	46.40	7.34	340.39	< 0.001
3	(27.093860 N, 116.250072 E) / 365.40 km	2020/6/1 to 2020/7/31	141	10.98	13.38	232.55	< 0.001
4	(46.977306 N, 113.669008 E) / 155.38 km	2021/8/1 to 2021/10/31	475	173.41	3.04	192.47	< 0.001
5	(43.544250 N, 80.993610 E) / < 1 km	2019/8/1 to 2019/8/31	65	10.79	6.13	62.97	< 0.001

Supplementary Table 7. Clusters determined by space-time permutation model for South East Asia data

Cluster	Coordinates / radius (km)	Time frame	Number of cases	Expected cases	LLR*	P-value
1	(15.491109 N, 103.887625 E) / 36.88 km	2021/3/1 to 2021/3/31	8562	399.27	399.27	< 0.001
2	(15.129047 N, 103.299063 E) / < 1 km	2021/7/1 to 2021/7/31	6295	203.18	15565.74	< 0.001
3	(16.083785 N, 103.728143 E) / 34.08 km	2021/4/1 to 2021/4/30	28620	8584.01	14921.05	< 0.001
4	(4.678979 S, 104.812898 E) / 942.57 km	2022/4/1 to 2023/3/31	3669	37.91	13161.23	< 0.001
5	(17.074690 N, 99.045670 E) / < 1 km	2021/11/1 to 2021/11/30	2517	16.87	10105.66	< 0.001
6	(14.943823 N, 103.060775 E) / < 1 km	2021/4/1 to 2021/4/30	9536	1938.64	7663.33	< 0.001
7	(15.638810 N, 100.464310 E) / 46.66 km	2022/3/1 to 2022/3/31	1635	9.83	6739.39	< 0.001
8	(16.547487 N, 104.629837 E) / 33.38 km	2021/5/1 to 2021/5/31	41525	22841.17	6580.42	< 0.001
9	(15.925540 N, 104.447450 E) / 27.42 km	2021/4/1 to 2021/4/30	9915	2503.20	6301.75	< 0.001
10	(16.327465 N, 102.207520 E) / 99.01 km	2021/5/1 to 2021/5/31	39964	22978.69	5497.34	< 0.001
11	(10.828294 N, 104.840030 E) / 396.62 km	2021/8/1 to 2021/10/31	1431	12.75	5339.05	< 0.001
12	(15.556740 N, 102.985880 E) / < 1 km	2021/6/1 to 2021/6/30	4642	625.20	5308.80	< 0.001
13	(13.231775 N, 99.813307 E) / 225.19 km	2021/6/1 to 2021/6/30	10682	3402.09	5005.68	< 0.001
14	(8.192840 N, 98.290950 E) / 359.80 km	2021/10/1 to 2022/3/31	1756	51.75	4488.01	< 0.001
15	(17.159282 N, 104.132534 E) / < 1 km	2021/6/1 to 2021/6/30	3525	474.76	4027.83	< 0.001

16	(14.590210 N, 103.137280 E) / < 1 km	2021/6/1 to 2021/6/30	2500	336.71	2854.33	< 0.001
17	(14.694110 N, 102.527350 E) / 62.72 km	2021/5/1 to 2021/5/31	20411	11953.87	2550.81	< 0.001
18	(17.299607 N, 103.447029 E) / 63.83 km	2021/5/1 to 2021/5/31	21012	12478.07	2505.25	< 0.001
19	(15.934613 N, 105.296460 E) / 65.40 km	2021/5/1 to 2021/5/31	15789	9328.44	1899.08	< 0.001

Supplementary Table 8. Clusters determined by space-time Poisson for South East Asia data

Cluster	Coordinates / radius (km)	Time frame	Number of cases	Expected cases	Relative risk	LLR*	P-value
1	(15.487439 N, 105.482810 E) / 180.29 km	2021/4/1 to 2021/6/30	87711	21809.32	4.98	62974.03	< 0.001
2	(6.098836 N, 102.378536 E) / 894.57 km	2021/5/1 to 2021/10/31	24652	7832.83	3.30	11849.24	< 0.001
